# Real-World Antiplatelet Use and Clinical Outcomes in Patients with Advanced Chronic Kidney Disease Following Acute Coronary Syndrome: A Descriptive Cohort Study

**DOI:** 10.3390/jcm15083167

**Published:** 2026-04-21

**Authors:** Lama Alfehaid, Eman Alzahrani, Amani Alsubaie, Majed Almutairi, Mansour Alomran, Saleh Alghadeer

**Affiliations:** 1 Department of Pharmacy Practice, College of Pharmacy, King Saud bin Abdulaziz University for Health Sciences, P.O. Box 3660, Riyadh 11481, Saudi Arabia; alsubaie10030@ksau-hs.edu.sa; 2 Pharmaceutical Care Department, King Abdulaziz Medical City, P.O. Box 22490, Riyadh 11426, Saudi Arabia; ph.emannnn@gmail.com (E.A.);; 3 King Abdullah International Medical Research Center, P.O. Box 3660, Riyadh 11481, Saudi Arabiaalghadeers@ksau-hs.edu.sa (S.A.); 4 College of Medicine, King Saud bin Abdulaziz University for Health Sciences, P.O. Box 3660, Riyadh 11481, Saudi Arabia; 5 King Abdulaziz Cardiac Center, King Abdulaziz Medical City, P.O. Box 22490, Riyadh 11426, Saudi Arabia

**Keywords:** acute coronary syndrome, chronic kidney disease, dual antiplatelet therapy, ticagrelor, clopidogrel, bleeding risk

## Abstract

**Background:** Patients with advanced chronic kidney disease (CKD) experience disproportionately high ischemic and bleeding risks following acute coronary syndrome (ACS), yet remain markedly underrepresented in randomized trials of antiplatelet therapy. Consequently, real-world data describing antiplatelet prescribing patterns and clinical outcomes in this population are limited. **Objectives:** To describe real-world antiplatelet use and 12-month clinical outcomes in patients with advanced CKD and end-stage renal disease (ESRD) following ACS. **Methods:** We conducted a single-center, retrospective cohort study including adults with advanced CKD (stage 4–5) or dialysis-dependent ESRD hospitalized with ACS and discharged on dual antiplatelet therapy. Baseline characteristics, revascularization strategies, and clinical outcomes were collected. Outcomes of interest included all-cause mortality, recurrent ischemic events (recurrent myocardial infarction, stroke or transient ischemic attack, or repeat revascularization), and bleeding events defined by Thrombolysis in Myocardial Infarction (TIMI) criteria over 12 months. All analyses were descriptive in nature. **Results:** A total of 222 patients were included; clopidogrel was prescribed in 96.0% of patients and ticagrelor in 4.0%. The cohort was elderly, highly comorbid, and predominantly dialysis-dependent. At 12 months, all-cause mortality occurred in approximately one-third of patients, recurrent ischemic events were frequent, and bleeding complications were common. Most bleeding events occurred in dialysis-dependent individuals. Outcomes among ticagrelor-treated patients are reported descriptively only due to the very small sample size. **Conclusions:** In this real-world cohort of patients with advanced CKD and ESRD following ACS, a substantial burden of mortality, recurrent ischemic events, and bleeding complications was observed, underscoring the narrow therapeutic window in this high-risk population. These findings are descriptive and hypothesis-generating, supporting the need for individualized antiplatelet strategies and prospective studies specifically enrolling patients with advanced CKD.

## 1. Introduction

Chronic kidney disease (CKD) is strongly associated with a high burden of coronary artery disease and a markedly increased risk of mortality and cardiovascular complications following acute coronary syndrome (ACS) [1]. Compared with individuals with preserved renal function, patients with CKD experience substantially worse short- and long-term outcomes after ACS, including higher rates of recurrent ischemic events, bleeding complications, and death. CKD is defined as abnormalities of kidney structure or function persisting for at least three months and is classified according to cause, glomerular filtration rate (GFR categories G1–G5), and albuminuria (categories A1–A3) [2].

ACS encompasses a spectrum of clinical presentations, including ST-elevation myocardial infarction (STEMI), non–ST–elevation myocardial infarction (NSTEMI), and unstable angina [3]. Its pathophysiology is driven by atherosclerotic plaque formation and disruption within the coronary arteries, leading to platelet activation, thrombus formation, and partial or complete coronary occlusion [4]. Contemporary registry data from the National Cardiovascular Data Registry–Acute Coronary Treatment and Intervention Outcomes Network (NCDR-ACTION) highlight the substantial overlap between CKD and ACS, with CKD present in approximately 30.5% of patients presenting with STEMI and 42.9% of those with NSTEMI [5].

Current guidelines from the American College of Cardiology/American Heart Association (ACC/AHA) and the European Society of Cardiology (ESC) identify dual antiplatelet therapy (DAPT) as a cornerstone of ACS management. DAPT consists of aspirin in combination with a P2Y12 receptor inhibitor. In patients undergoing percutaneous coronary intervention (PCI) and without contraindications, a more potent P2Y12 inhibitor, prasugrel or ticagrelor, is generally preferred over clopidogrel, whereas clopidogrel remains appropriate in patients with high bleeding risk, contraindications to potent agents, or intolerance [6,7].

Clopidogrel and prasugrel are prodrugs that require hepatic activation and irreversibly inhibit the P2Y12 receptor, whereas ticagrelor is a direct-acting, reversible P2Y12 inhibitor that provides more rapid and potent platelet inhibition [8]. Although intensified P2Y12 inhibition has been associated with improved ischemic outcomes in the general ACS population, bleeding remains the most frequent adverse effect of antiplatelet therapy. This concern is particularly pronounced in patients with CKD, who are intrinsically predisposed to bleeding due to uremia-related platelet dysfunction and comorbid conditions [9].

Despite their high cardiovascular risk, patients with advanced CKD, especially those with dialysis-dependent end-stage renal disease (ESRD), have been consistently underrepresented in the randomized trials that inform contemporary antiplatelet strategies. In the PLATO trial, which established the superiority of ticagrelor over clopidogrel in ACS, patients requiring dialysis were explicitly excluded [10,11]. Similarly, dialysis-dependent patients were excluded from the PEGASUS–TIMI 54 trial evaluating long-term secondary prevention [12], the THEMIS trial in patients with stable coronary artery disease and diabetes [13], and the TWILIGHT trial in the post-PCI setting [14]. Consequently, although these landmark trials have shaped guideline-directed antiplatelet therapy, their findings cannot be readily extrapolated to patients with advanced CKD or ESRD.

Importantly, patients with advanced CKD represent a distinct biological and pharmacological subgroup with unique alterations in hemostasis and drug response. Uremia is associated with impaired platelet adhesion, aggregation, and platelet–vessel wall interactions, contributing to increased bleeding risk [15,16]. At the same time, CKD is characterized by endothelial dysfunction, chronic inflammation, and enhanced prothrombotic signaling, which increase susceptibility to ischemic events [9,16]. In addition, altered drug metabolism and clearance may further influence the efficacy and safety of antiplatelet agents in this population [17]. These competing mechanisms create a narrow and complex therapeutic window that is not adequately captured in trials conducted in the general ACS population.

As a result, despite the widespread clinical use of clopidogrel and ticagrelor, the balance between ischemic benefit and bleeding risk associated with potent P2Y12 inhibition in patients with advanced CKD, particularly those requiring dialysis, remains uncertain. Consequently, there is a critical need for contemporary real-world data describing antiplatelet prescribing patterns and clinical outcomes in this vulnerable and understudied population. Given the persistent exclusion of patients with advanced CKD and ESRD from randomized clinical trials and the heterogeneity of real-world practice, the present study was designed as a descriptive cohort analysis rather than a comparative effectiveness study.

While prior studies have largely extrapolated findings from general ACS populations, our approach explicitly acknowledges the need for clinically meaningful interpretation within this high-risk subgroup. Therefore, beyond descriptive reporting, this study aims to contextualize real-world outcomes within the unique pathophysiological and pharmacological framework of advanced CKD.

Our objective was to characterize antiplatelet use, mortality, ischemic outcomes, and bleeding burden in a high-risk Saudi population to inform clinical decision-making and multidisciplinary care.

## 2. Materials and Methods

### 2.1. Study Design and Setting

This study was a single-center, retrospective observational cohort conducted at King Abdulaziz Medical City (KAMC) in Riyadh, Saudi Arabia, a tertiary care center providing advanced cardiovascular and renal services. The primary objective was to characterize real-world antiplatelet prescribing patterns and 12-month clinical outcomes among patients with advanced CKD and ESRD presenting with ACS. The study was intentionally designed as a descriptive cohort analysis and was not intended to evaluate comparative effectiveness between antiplatelet agents.

### 2.2. Study Subject

Adult patients aged 18 years or older who were admitted with a diagnosis of ACS, including ST-elevation myocardial infarction (STEMI), non–ST-elevation myocardial infarction (NSTEMI), or unstable angina, between January 2016 and January 2024 were screened for eligibility. Patients were included if they had advanced CKD, defined as stage 4 (estimated glomerular filtration rate [eGFR] 15–29 mL/min/1.73 m^2^) or stage 5 disease (eGFR < 15 mL/min/1.73 m^2^), or ESRD requiring chronic dialysis at the time of the index ACS event.

Eligible patients were required to have been discharged on either clopidogrel or ticagrelor as part of dual antiplatelet therapy following the index presentation. Patients were excluded if antiplatelet treatment data were unavailable, if follow-up information was completely missing, or if the diagnosis of ACS could not be confirmed based on clinical documentation. No exclusions were applied based on coronary anatomy or management strategy, and patients were included regardless of whether they were treated medically, underwent percutaneous coronary intervention (PCI), or had no obstructive coronary artery disease.

### 2.3. Data Collection

Clinical data were retrospectively extracted from electronic medical records using a standardized data abstraction process. Collected information included demographic characteristics (age, sex, body mass index), cardiovascular risk factors and comorbidities (diabetes mellitus, hypertension, dyslipidemia, prior bleeding history, heart failure, atrial fibrillation, venous thromboembolism, and smoking status), and renal parameters (baseline eGFR, CKD stage, and dialysis dependence). Smoking status was categorized as current smoker, former smoker, or non-smoker based on clinical documentation. When smoking status was not explicitly documented, it was classified as unknown, and only patients with clear documentation indicating no history of smoking were categorized as non-smokers.

Details of the index ACS presentation were recorded, including STEMI versus NSTEMI or unstable angina, coronary angiographic findings, number of diseased vessels, and revascularization strategy. Procedural variables included PCI performance, stent type, and anticoagulation use during hospitalization. Laboratory parameters, including hemoglobin, platelet count, lipid profile, and renal indices, were recorded at baseline and at 3, 6, and 12 months, when available.

### 2.4. Follow-Up

Patients were followed for 12 months from the date of the index ACS event. For time-to-event analyses, patients without a documented outcome were censored at 365 days. Patients with a documented date of death contributed observed follow-up time until death, while all remaining patients were censored at one year.

### 2.5. Study Outcomes

The primary outcomes of interest were descriptive measures of clinical effectiveness and safety. Effectiveness outcomes included all-cause mortality and the occurrence of non-fatal ischemic events, defined as recurrent myocardial infarction, stroke or transient ischemic attack, or repeat coronary revascularization within 12 months. Safety outcomes included any bleeding classified according to the Thrombolysis in Myocardial Infarction (TIMI) criteria [18], dyspnea requiring ticagrelor discontinuation, and clinically significant bradyarrhythmia requiring permanent pacemaker implantation.

Secondary outcomes included time to first non-fatal ischemic event and subgroup analyses stratified by dialysis status, comparing non-dialysis CKD stage 4 patients with dialysis-dependent ESRD patients. Kaplan–Meier survival estimates were used to describe all-cause mortality and event-free survival over the follow-up period for the overall cohort.

### 2.6. Confounding and Bias

Antiplatelet selection was determined by treating physicians based on clinical judgment, procedural strategy, and perceived ischemic and bleeding risk. As a result, treatment allocation was not random and is subject to selection bias and confounding by indication, particularly given the higher rate of PCI among ticagrelor-treated patients. Observed outcomes may therefore reflect differences in revascularization strategy rather than pharmacologic effects.

### 2.7. Statistical Analysis

All statistical analyses were performed using Stata version 19 (StataCorp., College Station, TX, USA). All analyses were descriptive in nature. Continuous variables are reported as means with standard deviations or medians with interquartile ranges, as appropriate. Categorical variables are summarized as counts and percentages.

Given the very small number of patients treated with ticagrelor, no formal statistical comparisons were performed between treatment groups, and no *p*-values are reported. Outcomes among antiplatelet subgroups are presented descriptively to illustrate real-world prescribing patterns and clinical burden rather than to infer comparative effectiveness or safety.

Kaplan–Meier analyses were performed for the overall cohort stratified by dialysis status rather than by antiplatelet agent, given the very small number of ticagrelor-treated patients. These analyses were exploratory and intended to provide clinical context rather than to test formal hypotheses.

### 2.8. Use of Artificial Intelligence (AI)

During the preparation of this manuscript, ChatGPT (OpenAI; GPT-5 series) was used solely for language editing and improving clarity. No AI tools were used for data collection, analysis, interpretation, or reference selection. All content was reviewed and verified by the authors, who take full responsibility for the accuracy and integrity of the manuscript.

## 3. Results

### 3.1. Study Population

**Figure 1:**
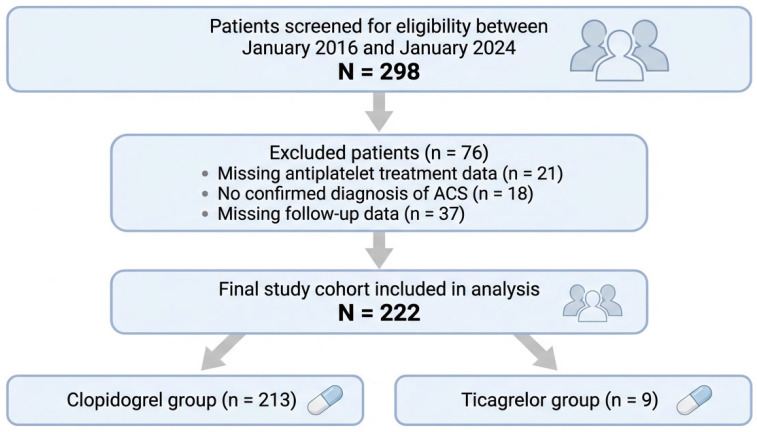
Study Flow Diagram of Patient Selection and Cohort Formation. Patients with advanced CKD and ESRD presenting with ACS were screened between 2016 and 2024. After applying exclusion criteria, 222 patients were included in the final analysis and stratified according to antiplatelet therapy at discharge.

A total of 298 patients were screened for eligibility between January 2016 and January 2024. Of these, 76 patients were excluded due to missing antiplatelet treatment data, unconfirmed diagnosis of acute coronary syndrome, or lack of follow-up information. The final study cohort consisted of 222 patients with advanced CKD or ESRD who met the inclusion criteria (Figure 1). Of these, 213 patients (96.0%) were discharged on clopidogrel, and 9 patients (4.0%) were discharged on ticagrelor.

The overall cohort was elderly and highly comorbid, with a substantial burden of cardiovascular disease and advanced renal dysfunction.

The mean age of the cohort was 64.8 ± 16.2 years, and the mean body mass index was 28.6 ± 6.5 kg/m^2^. Female patients comprised 41.4% of the study population. Diabetes mellitus (90.9%), hypertension (97.7%), and dyslipidemia (74.2%) were highly prevalent. A history of cardiovascular disease was present in 79.2% of patients, and heart failure was documented in 64.0%. Atrial fibrillation and venous thromboembolism were less frequent. Renal impairment was severe across the cohort. The mean estimated glomerular filtration rate (GFR) was 11.7 ± 8.6 mL/min/1.73 m^2^. Most patients were dialysis-dependent (82.4%), and the majority met criteria for ESRD (88.3%). Regarding smoking status, 9.0% of patients were current smokers, 13.5% were former smokers, 38.7% were non-smokers. A substantial proportion of patients had missing smoking data (48.8%), reflecting reliance on explicit clinical documentation, whereby smoking status was classified as unknown unless clearly specified.

Baseline demographic characteristics, comorbidities, cardiovascular history, renal function, and smoking status for the overall cohort are summarized in Table 1. Antiplatelet subgroup characteristics are presented descriptively to illustrate real-world prescribing patterns.

### 3.2. Follow-Up Duration

The median follow-up duration was 365 days (IQR 365–365), with a mean follow-up of 323.9 days. Most patients (≈83%) were censored at 1 year, consistent with the study’s fixed 12-month follow-up. Patients with a documented date of death contributed observed (uncensored) time, while all others were censored at 365 days.

### 3.3. Index Presentation and In-Hospital Management

At the index presentation, most patients were admitted with NSTEMI or unstable angina, while STEMI accounted for a smaller proportion of ACS presentations. Among patients discharged on clopidogrel, 9.9% had STEMI, whereas 22.2% of those discharged on ticagrelor had STEMI. Coronary angiography demonstrated a broad spectrum of coronary artery disease severity. A substantial proportion of clopidogrel-treated patients had no obstructive coronary lesions, whereas ticagrelor-treated patients more frequently demonstrated one- or two-vessel disease.

Angiographic and procedural characteristics are summarized in Table 2. Revascularization strategies varied across the cohort. Overall, PCI was performed in 23.9% of patients, with a higher rate among those discharged on ticagrelor. The absence of patients with ≥3 coronary lesions in the ticagrelor group likely reflects the very small sample size and variability in physician prescribing practices rather than a true clinical pattern. Among patients undergoing PCI, drug-eluting stents (DES) were the predominant stent type, while a small number underwent non-DES PCI (bare-metal stents or plain old balloon angioplasty). Heparin-based anticoagulation was the predominant intraprocedural strategy. The mean duration of intravenous anticoagulation during hospitalization was approximately 2–3 days. Dual antiplatelet therapy with aspirin was prescribed in all patients. Lipid parameters and other laboratory measures were collected at baseline and during follow-up and are summarized in Supplementary Table S1.

### 3.4. Clinical Outcomes

Overall clinical outcomes for the cohort are summarized in Table 3. During the 12-month follow-up, all-cause mortality occurred in 32.4% of patients. Non-fatal ischemic events, including recurrent myocardial infarction (MI), stroke or transient ischemic attack (TIA), and repeat coronary revascularization, were frequent. Recurrent MI and repeat revascularization accounted for the majority of ischemic events, while stroke/TIA events were infrequent.

Bleeding complications were common, with 16.4% of patients experiencing at least one TIMI bleeding event during follow-up. Most bleeding events occurred in dialysis-dependent patients. Outcomes among patients discharged on ticagrelor are reported descriptively only due to the very small sample size (n = 9) and should be interpreted cautiously.

Other adverse effects were infrequent in both treatment groups. Bradycardia was observed in 2 patients (0.9%) receiving clopidogrel and 1 patient (11.1%) receiving ticagrelor, while dyspnea occurred in 4 clopidogrel-treated patients (1.9%) and 1 ticagrelor-treated patient (11.1%). Pacemaker implantation was rare, occurring in 1 clopidogrel-treated patient (0.5%) and none in the ticagrelor group, as detailed in Supplementary Table S2.

### 3.5. Subgroup Analyses: Non-Dialysis CKD Stage 4 Patients

Among non-dialysis patients with CKD stage 4 (n = 41), a substantial burden of adverse clinical outcomes was observed during 12-month follow-up (Table 4). Non-fatal ischemic events, including recurrent myocardial infarction, stroke or transient ischemic attack, and repeat coronary revascularization, were frequent, and all-cause mortality remained high in this subgroup.

### 3.6. Subgroup Analyses: Dialysis-Dependent ESRD Patients

Among dialysis-dependent patients with ESRD (n = 181), the clinical burden during 12-month follow-up was even greater (Table 5). High rates of non-fatal ischemic events and all-cause mortality were observed, consistent with the advanced disease severity and comorbidity burden in this population.

### 3.7. Mortality and Causes of Death

Among patients who died during follow-up, infection-related causes were the most frequently documented contributors to mortality (23 cases). Other documented causes included acute MI, stroke, heart failure, renal failure, and procedure-related complications. However, cause-of-death information was unavailable for a substantial proportion of deceased patients. Detailed cause-of-death frequencies are provided in Supplementary Table S3.

### 3.8. Kaplan–Meier Analyses: Clinical Outcomes

**Figure 2:**
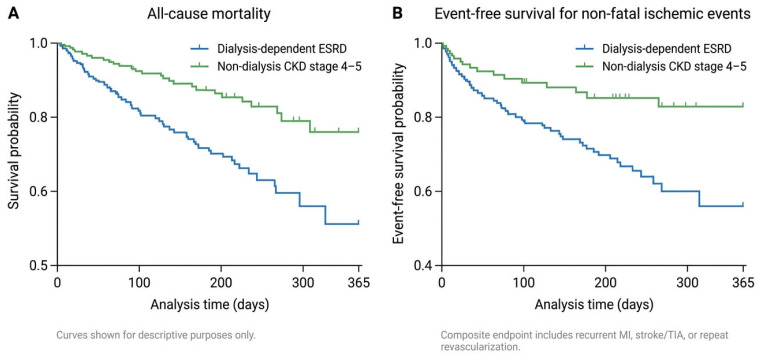
Kaplan–Meier analyses of clinical outcomes stratified by dialysis status. Panel (**A**). Kaplan–Meier estimates of all-cause mortality within 12 months following the index ACS event in patients with advanced CKD, stratified by dialysis dependence. Panel (**B**). Kaplan–Meier event-free survival for non-fatal ischemic events, defined as the first occurrence of recurrent myocardial infarction, stroke/transient ischemic attack, or repeat coronary revascularization within 12 months of the index ACS event, stratified by dialysis dependence. Curves are presented descriptively and are not intended to imply comparative treatment effects.

Figure 2 displays Kaplan–Meier estimates of clinical outcomes over 12 months following the index ACS event in patients with advanced CKD and ESRD, stratified by dialysis status.

Panel A shows Kaplan–Meier survival estimates for all-cause mortality. Survival declined progressively over the follow-up period, with lower survival observed among dialysis-dependent patients compared with those with non-dialysis CKD stage 4–5, reflecting a greater mortality burden in this subgroup.

Panel B shows Kaplan–Meier event-free survival for non-fatal ischemic events, defined as the first occurrence of recurrent myocardial infarction, stroke or transient ischemic attack, or repeat coronary revascularization within 12 months. Event-free survival declined over time in both groups, with dialysis-dependent patients demonstrating a higher cumulative incidence of adverse ischemic events.

These analyses are presented for descriptive purposes only and are intended to illustrate differences in outcome burden according to dialysis dependency rather than to imply comparative treatment effects.

## 4. Discussion

This single-center retrospective cohort study provides real-world insights into antiplatelet treatment patterns and 12-month clinical outcomes among patients with advanced CKD and ESRD following ACS. Several key observations emerge. Clopidogrel was predominantly used in routine clinical practice, whereas ticagrelor use was limited. Despite contemporary ACS management, patients with advanced CKD experienced a substantial burden of adverse outcomes, including approximately one-third of all-cause mortality at one year, frequent recurrent ischemic events, and a high incidence of bleeding complications [19]. These findings highlight the marked vulnerability of this population and underscore the narrow therapeutic window between ischemic protection and bleeding risk.

The high burden of both ischemic and bleeding events observed in this cohort is consistent with the complex hemostatic alterations described in advanced CKD. Rather than representing isolated risks, these competing mechanisms likely contribute to a narrowed therapeutic window in which the net clinical benefit of intensified antiplatelet therapy becomes less predictable [9,17,18,19,20], particularly in dialysis-dependent patients who were excluded from pivotal randomized trials [10,11]. This may partially explain why outcomes remain poor despite guideline-directed therapy and highlights the limitations of extrapolating evidence from the general ACS population to patients with advanced CKD [10,11].

Current guidelines from the ACC/AHA and ESC identify dual antiplatelet therapy (DAPT) as a cornerstone of ACS management; however, their applicability to patients with advanced CKD remains uncertain [6,7]. The Academic Research Consortium for High Bleeding Risk (ARC-HBR) classifies severe CKD and anemia as major bleeding risk criteria [16]. In our cohort, a large proportion of patients met multiple ARC-HBR criteria, reflecting a population in whom standard antiplatelet strategies may carry disproportionate risk. This underscores the need to interpret guideline recommendations cautiously in this subgroup.

Prescribing patterns observed in this study reflect real-world clinical decision-making rather than protocol-driven care. The predominant use of clopidogrel likely reflects its familiarity, lower cost, and perceived safety profile in patients at high bleeding risk. In contrast, ticagrelor was more frequently used in patients undergoing PCI, suggesting treatment selection based on perceived ischemic risk and procedural factors. These patterns should be interpreted cautiously, as the present study was not designed to compare antiplatelet strategies. Rather, the observed differences likely reflect underlying patient risk and clinical context. This introduces confounding by indication, whereby outcomes may be influenced by baseline risk and revascularization strategy rather than the pharmacologic effects of the antiplatelet agent itself.

Although this study was not designed to evaluate comparative effectiveness, several observations merit cautious interpretation. In the PLATO trial and its renal subgroup analyses, ticagrelor demonstrated superior efficacy compared with clopidogrel; however, dialysis-dependent patients were excluded, limiting applicability to our cohort [10,11].

In the present study, outcomes among ticagrelor-treated patients are reported descriptively due to the very small sample size and should be considered hypothesis-generating rather than confirmatory. Similarly, the absence of stroke events in the ticagrelor group should not be overinterpreted, given the limited number of patients and events.

The broader observational literature remains inconclusive. A meta-analysis by Burlacu et al. found no significant differences between ticagrelor and clopidogrel in dialysis-dependent patients across ischemic and bleeding outcomes [21]. These findings, together with our results, suggest that the incremental benefit of more potent P2Y12 inhibition in advanced CKD may be modest and difficult to disentangle from competing risks and residual confounding.

Bleeding risk remains a central concern in this population. Large population-based studies have demonstrated a substantially increased risk of major bleeding in patients with ESRD compared with those with preserved renal function [22]. In our cohort, TIMI bleeding events were common and occurred predominantly in dialysis-dependent individuals. This likely reflects the combined effects of uremic platelet dysfunction, anemia, advanced age, and repeated exposure to anticoagulation during dialysis. Consistent with these findings, time-to-event analyses demonstrated worse overall clinical outcomes among dialysis-dependent patients compared with those with non-dialysis CKD stage 4–5, underscoring dialysis status as a key determinant of risk in this population. Time-to-event analyses were intentionally stratified by dialysis status to reflect the dominant clinical risk gradient in this population.

Within this context, contemporary guidelines increasingly emphasize a risk-adapted approach to antiplatelet therapy. The 2025 ACC/AHA ACS guideline supports shorter DAPT duration with early aspirin discontinuation in high bleeding risk patients [6]. Trials such as SMART-CHOICE, STOPDAPT-2 ACS, and MASTER DAPT have demonstrated reduced bleeding without excess ischemic risk with abbreviated DAPT strategies [23,24,25]. However, patients with advanced CKD remain underrepresented in these trials, and extrapolation should be cautious.

While contemporary guidelines support abbreviated DAPT strategies in high bleeding risk populations, our study does not evaluate DAPT duration, and these approaches should not be inferred from the present data.

Taken together, these findings highlight the importance of individualized antiplatelet therapy in patients with advanced CKD following ACS. Clinical decision-making should integrate ischemic and bleeding risks, dialysis status, comorbid conditions, and procedural factors. From a clinical pharmacy perspective, these results support the role of multidisciplinary care, including medication optimization, bleeding surveillance, and coordination with dialysis schedules.

Several limitations should be acknowledged. First, the retrospective, single-center design limits generalizability and precludes causal inference. Second, residual confounding is inherent to observational studies and is particularly relevant given physician-driven antiplatelet selection. Third, significant baseline imbalances were present, including a substantially higher rate of PCI among ticagrelor-treated patients, as well as incomplete data for certain variables, such as smoking status, which was undocumented in a large proportion of patients. Smoking classification relied on explicit clinical documentation and may have introduced misclassification bias.

Fourth, the very small number of patients treated with ticagrelor limits statistical power and precludes meaningful comparative or regression analyses, increasing the risk of model instability and unreliable estimates. Fifth, differences in revascularization strategy may have independently influenced clinical outcomes. Sixth, the study period spanned several years during which clinical practice guidelines and prescribing patterns evolved; however, temporal trends in antiplatelet use were not formally analyzed, which may limit interpretation of treatment patterns over time. Additionally, medication adherence after discharge could not be reliably assessed, and cause-of-death data were incomplete in a substantial proportion of cases. These factors limit interpretation and reinforce the descriptive nature of the analysis.

## 5. Conclusions

This study provides contemporary real-world data on antiplatelet prescribing patterns and 12-month clinical outcomes among patients with advanced CKD and ESRD following ACS. Despite guideline-directed therapy, this population experienced a substantial burden of all-cause mortality, recurrent ischemic events, and bleeding complications, underscoring the narrow therapeutic window between ischemic protection and bleeding risk in advanced CKD. Importantly, this cohort includes a large proportion of dialysis-dependent patients who are consistently underrepresented in randomized clinical trials, offering clinically relevant insights from a high-risk population.

The high prevalence of ARC-HBR features, combined with frequent TIMI bleeding events, highlights the need for individualized antiplatelet strategies that carefully balance ischemic and bleeding risks while accounting for renal function, anemia, dialysis status, and revascularization approach. Within the context of contemporary risk-adapted frameworks, these findings support consideration of tailored antiplatelet approaches; however, they should not be interpreted as evidence of comparative effectiveness.

Importantly, the findings of this study are descriptive and hypothesis-generating, reflecting real-world practice in a high-risk population with substantial baseline imbalances and limited representation of potent P2Y12 inhibitor use. Prospective, adequately powered studies specifically enrolling patients with advanced CKD are urgently needed to better define optimal antiplatelet strategies and improve clinical outcomes in this vulnerable population.

## Figures and Tables

**Table 1:** Baseline Characteristics.

Variable	Clopidogrel (n = 213)	Ticagrelor (n = 9)
**Demographics**		
Age, years	64.8 ± 16.5	63.9 ± 6.5
BMI, kg/m^2^	28.5 ± 6.6	31.4 ± 4.1
Female sex, n (%)	87 (40.8)	5 (55.6)
**Comorbidities**		
Diabetes mellitus, n (%)	193 (90.6)	9 (100.0)
Hypertension, n (%)	209 (98.1)	8 (88.9)
Dyslipidemia, n (%)	155/212 (73.1)	9 (100.0)
History of bleeding, n (%)	25 (11.7)	2 (22.2)
**Cardiovascular Disease History**		
Cardiovascular disease, n (%)	169 (79.3)	7 (77.8)
Heart failure, n (%)	139 (65.3)	3 (33.3)
Atrial fibrillation, n (%)	37 (17.4)	0 (0.0)
Venous thromboembolism, n (%)	15 (7.0)	1 (11.1)
**Renal Function**		
GFR, mL/min/1.73 m^2^	11.6 ± 8.5	13.0 ± 9.5
On dialysis, n (%)	176 (82.6)	7 (77.8)
ESRD (CKD stage 5), n (%)	184/207 (88.9)	7 (77.8)
**Smoking History**		
Current smoker, n (%)	19 (8.9)	1 (11.1)
Former smoker, n (%)	9 (4.2)	1 (11.1)
Non-smoker, n (%)	83 (39.0)	3 (33.3)
Unknown, n (%)	102 (47.9)	4 (44.4)

Smoking status was classified based on explicit clinical documentation; patients without documented smoking status were categorized as unknown.

**Table 2:** Current diagnosis and management according to the antiplatelet group.

Variable	Clopidogrel (n = 213)	Ticagrelor (n = 9)
STEMI as index event	21/213 (9.9%)	2/9 (22.2%)
NSTEMI/UA as index event	192/213 (90.1%)	7/9 (77.8%)
0 coronary lesions	135/213 (63.4%)	2/9 (22.2%)
1 coronary lesion	39/213 (18.3%)	3/9 (33.3%)
2 coronary lesions	28/213 (13.1%)	4/9 (44.4%)
≥3 coronary lesions	11/213 (5.2%)	0/9 (0.0%)
Any PCI performed	46/213 (21.6%)	7/9 (77.8%)
DES PCI (among all)	45/213 (21.1%)	5/9 (55.6%)
Non-DES PCI (BMS or POBA, among all)	0/213 (0.0%)	2/9 (22.2%)
Heparin-based anticoagulation	212/213 (99.5%)	9/9 (100.0%)
Duration of IV anticoagulant, days	2.9 ± 2.2 (n = 212)	2.0 ± 1.4 (n = 9)

**Table 3:** Efficacy and Safety Outcomes.

Outcome	Clopidogrel (n = 213)	Ticagrelor (n = 9)
Composite efficacy outcome	106/213 (49.8%)	4/9 (44.4%)
All-cause death	69/213 (32.4%)	2/9 (22.2%)
Recurrent MI	49/213 (23.0%)	2/9 (22.2%)
Stroke/TIA	5/213 (2.3%)	0/9 (0.0%)
Repeat revascularization	17/213 (8.0%)	2/9 (22.2%)
Any TIMI bleeding	35/213 (16.4%)	1/9 (11.1%)

**Table 4:** Outcomes in Dialysis Patients (ESRD).

Outcome	Clopidogrel (n = 37)	Ticagrelor (n = 4)
Composite outcome	15/37 (40.5%)	2/4 (50.0%)
All-cause death	9/37 (24.3%)	1/4 (25.0%)
Recurrent MI	6/37 (16.2%)	1/4 (25.0%)
Stroke or TIA	1/37 (2.7%)	0/4 (0.0%)
Repeat revascularization	2/37 (5.4%)	1/4 (25.0%)
Any TIMI bleeding	7/37 (18.9%)	1/4 (25.0%)

Note: Ticagrelor subgroup is very small (n = 5); results are descriptive and not inferential.

**Table 5:** Outcomes in Dialysis Patients (ESRD).

Outcome	Clopidogrel (n = 176)	Ticagrelor (n = 5)
Composite outcome	91/176 (51.7%)	2/5 (40.0%)
All-cause death	60/176 (34.1%)	1/5 (20.0%)
Recurrent MI	43/176 (24.4%)	1/5 (20.0%)
Stroke or TIA	4/176 (2.3%)	0/5 (0.0%)
Repeat revascularization	15/176 (8.5%)	1/5 (20.0%)
Any TIMI bleeding	28/176 (15.9%)	0/5 (0.0%)

Note: Ticagrelor subgroup is very small (n = 5); results are descriptive and not inferential.

## Data Availability

The data supporting the findings of this study are not publicly available due to patient confidentiality concerns and institutional restrictions imposed by King Abdullah International Medical Research Center. Data may be available from the corresponding author upon reasonable request and subject to institutional approval.

## Supplementary Materials

The following supporting information can be downloaded at: https://www.mdpi.com/article/10.3390/jcm15083167/s1, Table S1: Laboratory values at baseline, 3, 6, and 12 months according to the antiplatelet group; Table S2: Adverse Effects by Antiplatelet Group; Table S3: Outcomes in Non-dialysis Patients; Table S4: Causes of Death.

## Abbreviations

ACS	Acute Coronary Syndrome
ARC-HBR	Academic Research Consortium for High Bleeding Risk
CKD	Chronic Kidney Disease
DAPT	Dual Antiplatelet Therapy
eGFR	Estimated Glomerular Filtration Rate
ESRD	End-Stage Renal Disease
IRB	Institutional Review Board
KAIMRC	King Abdullah International Medical Research Center
MI	Myocardial Infarction
NSTEMI	Non-ST-Elevation Myocardial Infarction
PCI	Percutaneous Coronary Intervention
STEMI	ST-Elevation Myocardial Infarction
TIA	Transient Ischemic Attack
TIMI	Thrombolysis in Myocardial Infarction
