# Supplementary material for: Real-World Antiplatelet Use and Clinical Outcomes in Patients with Advanced Chronic Kidney Disease Following Acute Coronary Syndrome: A Descriptive Cohort Study
Source: J Clin Med. 2026 Apr 21;15(8):3167. doi: 10.3390/jcm15083167 (PMC13118201; doi:10.3390/jcm15083167)
Supplement: Supplementary file 1 [file jcm-15-03167-s001.zip › jcm-4212429-supplementary.pdf]

Supplementary Materials

Table S1. Laboratory values at baseline, 3, 6, and 12 months according to the antiplatelet group

Analyte / Timepoint	Clopidogrel	Ticagrelor
**Hemoglobin – Baseline**	112.1 ± 18.3 (n=213)	108.1 ± 13.7 (n=9)
Hemoglobin – 3 months	112.4 ± 20.4 (n=154)	111.0 ± 15.9 (n=6)
Hemoglobin – 6 months	112.8 ± 20.2 (n=147)	110.8 ± 14.2 (n=5)
Hemoglobin – 12 months	111.9 ± 16.5 (n=121)	106.9 ± 15.2 (n=4)
**Platelets – Baseline (×10⁹/L)**	237.2 ± 91.7 (n=213)	210.8 ± 63.1 (n=9)
Platelets – 3 months	231.9 ± 86.4 (n=154)	227.7 ± 56.7 (n=6)
Platelets – 6 months	223.2 ± 80.9 (n=146)	229.4 ± 69.6 (n=5)
Platelets – 12 months	221.4 ± 85.7 (n=121)	220.5 ± 59.0 (n=4)
**GFR – Baseline (mL/min/1.73m²)**	11.6 ± 8.5 (n=213)	13.0 ± 9.5 (n=9)
**Total cholesterol – Baseline (mmol/L)**	3.7 ± 1.2 (n=213)	4.3 ± 1.2 (n=9)
Total cholesterol – 3 months	7.8 ± 42.7 (n=93)	3.4 ± 1.0 (n=5)
Total cholesterol – 6 months	6.3 ± 26.7 (n=86)	3.8 ± 1.7 (n=5)
Total cholesterol – 12 months	3.5 ± 1.1 (n=65)	2.8 ± 0.6 (n=4)
**LDL – Baseline (mmol/L)**	2.1 ± 1.0 (n=213)	2.9 ± 1.0 (n=9)
LDL – 3 months	4.9 ± 21.1 (n=51)	1.8 ± 1.2 (n=4)
LDL – 6 months	4.0 ± 17.4 (n=45)	1.6 ± 1.9 (n=3)
LDL – 12 months	2.0 ± 0.8 (n=33)	0.9 ± 0.3 (n=2)
**HDL – Baseline (mmol/L)**	0.9 ± 0.3 (n=213)	0.9 ± 0.2 (n=9)
HDL – 3 months	0.9 ± 0.3 (n=54)	1.0 ± 0.2 (n=4)
HDL – 6 months	0.9 ± 0.3 (n=45)	1.1 ± 0.2 (n=3)
HDL – 12 months	0.9 ± 0.3 (n=34)	1.1 ± 0.3 (n=2)
**Triglycerides – Baseline (mmol/L)**	1.6 ± 0.9 (n=213)	1.6 ± 0.6 (n=9)
Triglycerides – 3 months	1.6 ± 0.9 (n=93)	1.1 ± 0.3 (n=4)
Triglycerides – 6 months	1.6 ± 0.9 (n=86)	1.3 ± 0.1 (n=3)
Triglycerides – 12 months	1.7 ± 1.2 (n=65)	1.3 ± 0.5 (n=2)

Table S2. Adverse Effects by Antiplatelet Group

Side effect	Clopidogrel (n=213)	Ticagrelor (n=9)
**Bradycardia**	2/213 (0.9%)	1/9 (11.1%)
**Pacemaker implantation**	1/213 (0.5%)	0/9 (0.0%)
**Dyspnea**	4/213 (1.9%)	1/9 (11.1%)
**No reported side effect**	206/213 (96.7%)	7/9 (77.8%)

Table S3. Outcomes in Non-dialysis Patients

Outcome	Clopidogrel (n=37)	Ticagrelor (n=4)
Composite outcome	15/37 (40.5%)	2/4 (50.0%)
All-cause death	9/37 (24.3%)	1/4 (25.0%)
Recurrent MI	6/37 (16.2%)	1/4 (25.0%)
Stroke or TIA	1/37 (2.7%)	0/4 (0.0%)

Repeat revascularization	2/37 (5.4%)	1/4 (25.0%)
Any TIMI bleeding	7/37 (18.9%)	1/4 (25.0%)

Note: Ticagrelor subgroup is very small (n = 5); results are descriptive and not inferential.

Table S4. Causes of Death

Cause of Death	N
Infection (sepsis, pneumonia, etc.)	23
Other/unspecified	20
Myocardial infarction	7
Fatal arrhythmia	2
Stroke	1
Renal failure	1
Death during/after procedure	1
Heart failure (low-output)	1
Cause not documented	166
